# Autologous hematopoietic stem cell transplantation improves long-term survival—data from a national registry

**DOI:** 10.1186/s13075-022-02948-x

**Published:** 2022-11-22

**Authors:** Norbert Blank, Marc Schmalzing, Pia Moinzadeh, Max Oberste, Elise Siegert, Ulf Müller-Ladner, Gabriela Riemekasten, Claudia Günther, Ina Kötter, Gabriele Zeidler, Christiane Pfeiffer, Aaron Juche, Ilona Jandova, Jan Ehrchen, Laura Susok, Tim Schmeiser, Cord Sunderkötter, Jörg H. W. Distler, Margitta Worm, Alexander Kreuter, Gernot Keyßer, Hanns-Martin Lorenz, Thomas Krieg, Nicolas Hunzelmann, Jörg Henes, on behalf of the German Network for Systemic Sclerosis

**Affiliations:** 1 Internal Medicine 5, Division of Hematology, Oncology and Rheumatology, Department of Rheumatology, University Hospital Heidelberg, Im Neuenheimer Feld 410, 69120 Heidelberg, Germany; 2 Department of Rheumatology/Clinical Immunology, University Hospital Wuerzburg, Wuerzburg, Germany; 3 Department of Dermatology and Venereology, University Hospital Cologne, Cologne, Germany; 4 Institute of Medical Statistics and Computational Biology, Faculty of Medicine, University of Cologne, Cologne, Germany; 5 Department of Rheumatology, Charité Universitätsmedizin Berlin, Berlin, Germany; 6 Campus Kerckhoff, Department of Rheumatology and Clinical Immunology, Justus Liebig University Giessen, Bad Nauheim, Germany; 7 Department of Rheumatology, University Medical Center-UKSH, Luebeck, Germany; 8 Department of Dermatology, University Hospital Carl Gustav Carus, Dresden, Germany; 9 Department of Rheumatology and Immunology, University Hospital, Hamburg-Eppendorf and Rheumatology Hospital Bad Bramstedt, Bad Bramstedt, Germany; 10 Department of Rheumatology, Johanniter-Krankenhaus im Flaeming Treuenbrietzen, Treuenbrietzen, Germany; 11 Department of Dermatology, University Hospital Munich, Munich, Germany; 12 Department of Rheumatology, Immanuel Krankenhaus Berlin-Buch, Berlin, Germany; 13 Department of Rheumatology, University Hospital Freiburg, Freiburg im Breisgau, Germany; 14 Department of Dermatology and Venerology, University Hospital Muenster, Muenster, Germany; 15 Department of Dermatology and Venereology, Ruhr-University-Bochum, Bochum, Germany; 16 Department of Dermatology, St. Josef Hospital Wuppertal, Wuppertal, Germany; 17 Dermatology, University Hospital Halle, Halle, Germany; 18 Department of Rheumatology, University Hospital Erlangen, Erlangen, Germany; 19 Department of Dermatology and Allergology, Charité Universitätsmedizin Berlin, Berlin, Germany; 20 Allergology, HELIOS St. Elisabeth Hospital Oberhausen, University Witten-Herdecke, Witten, Germany; 21 Rheumatology, University Hospital Halle, Halle, Germany; 22 Centre for Interdisciplinary Clinical Immunology, Rheumatology and Auto-inflammatory Diseases and Department of Internal Medicine II (Oncology, Hematology, Immunology, Rheumatology), University Hospital Tuebingen, Tuebingen, Germany

**Keywords:** Scleroderma, Systemic sclerosis, Autologous hematopoietic stem cell transplantation, German network for systemic scleroderma

## Abstract

**Background:**

Current recommendations on the management of systemic sclerosis (SSc) suggest that autologous hematopoietic stem cell therapy (HSCT) can be a rescue therapy for patients with rapidly progressive SSc.

**Objectives:**

To assess the safety and efficacy of HSCT for patients with SSc and to compare these with non-HSCT patients in a control cohort with adjusted risk factors.

**Methods:**

A retrospective analysis of data from the multicentric German network for systemic scleroderma (DNSS) with 5000 patients with SSc. Control groups consisted of all patients with diffuse cutaneous (dc)-SSc (group A) and an adjusted high-risk cohort of male patients with Scl70-positive dc-SSc (group B).

**Results:**

Eighty SSc patients received an HSCT 4.1 ± 4.8 years after SSc diagnosis. Among them, 86.3% had dc-SSc, 43.5% were males, and 71.3% were positive for Scl70 antibodies. The control group A (*n*=1513) showed a significant underrepresentation of these risk factors for mortality. When the survival of the control group B (*n*=240) was compared with the HSCT group, a lower mortality of the latter was observed instead. Within 5 years after HSCT, we observed an improvement of the mRSS from 17.6 ± 11.5 to 11.0 ± 8.5 (*p*=0.001) and a stabilization of the DLCO. We did not see differences in transplant-related mortality between patients who received HSCT within 3 years after SSc diagnosis or later.

**Conclusion:**

Our analysis of real-life data show that the distribution of risk factors for mortality is critical when HSCT cohorts are compared with non-HSCT control groups.

## Key messages

1. HSCT improves long-term survival in patients with progressive SSc
2. Real-life data form the German Network of Systemic Scleroderma (DNSS) cohort
3. Risk factors for mortality are crucial to assess survival in HSCT and control groups

## Introduction

Systemic sclerosis (SSc) is a complex multisystem autoimmune disease, which affects the skin and internal organs, such as the lung, heart, gastrointestinal tract, musculoskeletal system, and kidneys [1]. Until today, effective treatment options are very limited. Immunosuppression, especially in early disease (<2 years from first non-Raynaud phenomenon), is recommended using methotrexate or cyclophosphamide [2]. A randomized controlled trial showed equivalent efficacy of mycophenolate and cyclophosphamide in patients with SSc-associated interstitial lung disease (ILD) [3]. Nintedanib was effective in reducing the progression of SSc ILD as an antifibrotic treatment [4]. Very recently, tocilizumab showed some benefit with regard to the lung involvement but not for the skin sclerosis [5]. Furthermore, an observational study of rituximab showed some benefit for the skin sclerosis but not for the lung function parameters [6]. Currently, no biologic treatment option has been approved for SSc skin sclerosis.

Risk factors for the progressive disease have been identified during the last years, and male patients with early diffuse cutaneous skin manifestations and anti-topoisomerase I (Scl70) antibodies are at high risk for disease-related mortality [7, 8].

Autologous hematopoietic stem cell transplantation (HSCT) is a very effective and meanwhile well-established treatment option for SSc patients with a severe course of the disease. Therefore, HSCT was included in the current EULAR management guidelines [2]. Three randomized controlled trials proved its superiority over standard treatment with cyclophosphamide pulses in severe cases [9–11], and a recent non-interventional trial showed its effectiveness also in a real-life setting [12]. The aim of the treatment strategy is improvement of long-term survival by eliminating the auto-aggressive immune system using high-dose chemotherapy in combination with lymphoablative antibodies or myeloablative radiation. Major points of criticism are side effects, especially the treatment-related mortality (TRM) between 5 and 10% [9–12]. Therefore, it is very important to discuss and compare the risk for disease-related mortality with TRM. To investigate long-term survival in a subgroup of patients with a high risk for disease-related mortality, we compared data of patients with and without HSCT from the registry of the German Network for Systemic Scleroderma.

## Methods

The patient registry of the German Network for Systemic Scleroderma (DNSS) was founded in 2003 and up to now includes more than 5000 patients. The network involves more than 40 clinical centers with different subspecialties including rheumatologists, dermatologists, pulmonologists, and nephrologists. The Ethics Committee of the coordinating center, i.e., the Dept. of Dermatology at the University Hospital Cologne, approved the patient information and consent form of the DNSS registry (approval number 04-037), which was used by all participating centers to receive the approval of their local ethics committees prior to registering patients. To participate in the registry, all patients obtained written informed consent. The 4-page disease- and organ-specific questionnaire collects clinical data to determine the current disease status with information on gender, age, disease onset, and auto-antibodies and therapies longitudinally over time [13, 14].

### Definition of included SSc subsets

Patients met the 2013 American College of Rheumatology (ACR)/European League against Rheumatism collaborative initiative criteria for systemic sclerosis [15]. The SSc subtype was based on the classification criteria established by LeRoy et al. [16, 17]. Follow-up visits were performed at least once per year. In the present analysis, we focused on male patients with dc-SSc and positive Scl70 antibodies as a control group.

### Assessment of skin sclerosis

The modified Rodnan skin sclerosis (mRSS) score was used to quantify skin sclerosis. Repetitive determination of the mRSS score was preferentially performed by the same trained investigator to minimize variations.

### Definition of heart involvement

Heart involvement was defined as the presence of elevated troponin T, pericardial effusion, pathologic changes in cardiac MRI, ventricular extrasystoles > Lown 3, or the presence of endomyocardial fibrosis in a biopsy specimen.

### HSCT protocols

Patients received HSCT between 1997 and 2018. Cyclophosphamide 2×2g/m^2^ was used for mobilization chemotherapy until 2012 and 2×1g/m^2^ after 2012 with equally sufficient hematopoietic stem cell (HSC) harvest gains. All patients received G-CSF from day +4 until the end of the HSC harvest. Some transplants were manipulated by enhancing HSC using CD34-loaded columns and/or anti-CD3 and anti-CD19 monoclonal antibodies to minimize residual autoreactive T- and B-cells in the transplant. In any case, the goal was to receive 2.5×10^6^ CD34+ HSC per kg body weight at the end of the harvesting procedure. Conditioning chemotherapy was performed by using cyclophosphamide 200 mg/kg, melphalan 140 mg/m^2^, or a combination of cyclophosphamide 100 mg/kg and thiotepa 10 mg/kg in patients with cardiac involvement. After the hematologic reconstitution, a prophylaxis with cotrimoxazole, nystatin, and aciclovir was continued for 3 to 6 months to prevent pneumocystis, candida, and herpes virus reactivation. We did not recognize any significant differences between patients and outcomes and between the several protocols.

### Statistic analyses

The DNSS data registry was analyzed for patients who were treated with HSCT. Two control groups of patients with dc-SSc were used as references. Control group A comprised patients with dc-SSc and moderate risk for mortality. Control group B comprised male patients with dc-SSc who were positive for anti-Scl70 and had a high risk for mortality. To compare demographic and serological parameters, we used data of the initial visit. To compare organ manifestations and HSCT treatment, we used follow-up data of all visits.

The overall survival was assessed using Kaplan-Meier analyses and compared between the HSCT group and control groups using log-rank tests. Results are presented as Kaplan-Meier curves and the corresponding 95% confidence intervals were compared 5, 10, and 15 years after SSc onset which is the time of the first non-Raynaud manifestation.

To compare the disease progression within the HSCT and control cohorts, the mRSS, diffusion capacity of the lungs (DLCO-SB), and the body mass index (BMI) were analyzed after 1, 3, and 5 years and tested for statistical significance using separate dependent *t*-tests.

To investigate mortality risk factors for HSCT-treated patients, we compared patients younger than age 50 years with older patients and SSc disease duration of less than 3 years between the first non-Raynaud symptom and longer SSc disease duration.

For group comparisons, we used Pearson’s chi-square test (qualitative data) and the Kruskal-Wallis test (quantitative data). All reported *p* values are 2-sided and *p* values of < 0.05 were considered statistically significant. As the analyses were regarded as explorative, we did not adjust for multiple testing. Calculations and figures were carried out using SPSS (23.0.0.3 64-Bit, IBM Corp., Armonk, NY, USA) and Excel (Microsoft Corp., Redmond, WA, USA), as well as R (version 3.4.0, R Foundation for Statistical Computing, Vienna, Austria).

## Results

### Epidemiology and definition of control groups

Between 2003 and 2019, more than 5000 patients with systemic sclerosis (SSc) were recruited to the German Network for Systemic Scleroderma (DNSS). Within this cohort, 80 patients (1.6%) were treated with high-dose chemotherapy followed by autologous hematopoietic stem cell transplantation (HSCT) between 1997 and 2018. Indications for HSCT were an mRSS > 10 (68%), presence of ILD (66%), heart involvement (24%), or combinations of poor risk factors. Nine patients received HSCT before the year 2003 and before they entered the DNSS registry. The mean age at SSc diagnosis, HSCT, and DNSS baseline were 38.3 years, 43.0 years, and 44.3 years, respectively (Table 1). The mean time period (± SD) between the age at SSc diagnosis and the age at HSCT was 4.1 ± 4.8 years. Sixty-nine patients (86.3%) in the HSCT group had a diffuse cutaneous (dc-SSc) form of SSc. Therefore, dc-SSc patients who did not receive HSCT were considered as control group A. Within the DNSS registry, 1513 patients with dc-SSC were identified as control group A (Table 1). However, further analyses indicated statistically significant differences between the HSCT and control group A. HSCT patients were younger at dc-SSc diagnosis and younger at DNSS entry at baseline (Table 1). In the HSCT group, more patients were male (43.8% versus 26.8%), had anti-Scl70 antibodies (71.3% versus 53.7%), and had a lower body mass index (BMI 22.8 kg/m^2^ versus 24.3 kg/m^2^) compared to control group A (Table 1). These findings confirm that SSc patients had a poor prognosis before they entered the HSCT program. The epidemiologic findings in Table 1 also suggest that all patients with dc-SSc (control group A) might be not an adequate control group for HSCT.

**Table 1:** The epidemiology of HSCT, dc-SSc control group A, and male+Scl70+ dc-SSc control group B showed the prevalence of risk factors for progressive SSc in all three cohorts at baseline

**Baseline DNSS visit**		**HSCT**	**all dcSSccontrols A**	**p**	**dcSSc+ Scl70+ male controls B**	**p**
Patient number (N)		80	1513	---	240	---
Age at SSc diagnosis (mean ± SD)		38.3 ± 11.7	46.6 ± 14.2	<0.001	46.5 ± 12.1	<0.001
Age at HSCT		43.0 ± 10.9				
Time diagnosis to HSCT		4.1 ± 4.8				
Age at DNSS baseline		44.3 ± 10.8	53.3 ± 13.6	<0.001	50.3 ± 12.0	0.002
Clinical course	Limited	10 (12.5)	0		0	
	diffuse	69 (86.3)	1433 (94.7)		240 (100.0)	
	other	1 ( 1.3)	0		0	
Male gender		35 (43.8)	406 (26.8)	0.003	240 (100.0)	
ANA positive (>1:80, N (%))		73 (91.3)	1236 (81.7)	1.0	240 (100.0)	
Anti Scl70 / topoisomerase		57 (71.3)	812 (53.7)	0.034	240 (100.0)	
Body mass index (kg/m^2^)		22.8 ± 4.2	24.3 ± 4.6	0.015	25.0 ± 4.5	0.001

Therefore, we decided to include a second control group comprising patients with dc-SSc and Scl70 antibodies and male gender (control group B). Among control group A, 240 patients were identified for the more restrictively defined control group B (Table 1). Although control group B was defined as a more homogenous control group with high-risk features, significant differences regarding the age at SSc diagnosis, age at DNSS entry, ANA and Scl70 positivity, and BMI persisted when compared with the HSCT group (Table 1).

### Skin sclerosis at baseline

Quantification of the skin sclerosis with the modified Rodnan skin score (mRSS) was 17.7 on average in the HSCT group, 15.6 in the control group A, and 17.2 in the high-risk control group B (Table 2). We found a significantly lower number of patients with severe skin sclerosis (mRSS > 20) in control group A (28.2%) compared to the HSCT group (41.0%) and control group B (32.1%).

**Table 2:** The prevalence and the extent of skin sclerosis, lung, and heart involvement at the first DNSS visit were indicated for HSCT and control groups A and B

**Baseline DNSS visit**		**HSCT**	**all dc-SSc controls A**	**p**	**dc-SSc+ Scl70+ male controls B**	**p**
Patient number (N)		80	1513	---	240	
Patient number valid n (%)		78 (97.5)	1241 (82.0)	---	218 (90.3)	
mRSS (mean ± SD)		17.7 ± 11.7	15.6 ± 9.9	0.156	17.2 ± 9.7	0.860
mRSS < 10 (N (%))		25 (32.1)	423 (34.1)	0.805	59 (27.1)	0.464
mRSS > 10		53 (68.0)	818 (65.9)		159 (72.9)	
mRSS > 15		42 (53.8)	557 (44.9)	0.128	115 (52.8)	0.895
mRSS > 20		32 (41.0)	350 (28.2)	0.020	70 (32.1)	0.167
Lung involvement	PAH only	0	58 ( 3.8)	0.186	9 (3.8)	0.288
	ILD only	42 (52.5)	658 (43.5)		113 (47.1)	
	PAH + ILD	11 (13.8)	171 (11.3)		28 (11.7)	
	none	26 (32.5)	522 (34.5)		87 (36.3)	
DLCO SB valid data (N (%))		60 (75.0)	829 (54.8)	0.001	163 (67.9)	0.147
DLCO SB (%, mean ± SD)		53.9 ± 17.9	63.2 ± 21.9	0.006	59.1 ± 21.3	0.124
DLCO SB <75% (N (%))		53 (88.3)	633 (76.4)		129 (79.1)	
Heart involvement (N (%))		19 (23.8)	243 (16.1)	0.131	52 (21.7)	0.756

### Interstitial lung disease and heart involvement at baseline

ILD was observed in 52.5% of the HSCT group, compared to 43.5% in control group A and 47.1% in control group B (Table 2) confirming that patients in the HSCT group were negatively selected for rapidly progressive disease. Since PAH has a well-known high risk for mortality, patients with unstable or moderate to severe PAH should not be considered for HSCT. Eleven patients with mild and stable PH associated with ILD (13.8%) were identified in the HSCT group compared with 11.3% in control group A and 11.7% in control group B (Table 2). The single-breath diffusion lung capacity for carbon monoxide (DLCO-SB) was more impaired in the HSCT group (53.9%) compared with control group A (63.2%, Table 2).

Heart involvement was documented in 23.4% in the HSCT group, 16.1% in control group A, and 21.7% in control group B, which confirms that control group B is a better comparator for patients in the HSCT group.

### HSC mobilization and conditioning chemotherapy

Between 1997 and 2012, 37 patients received mobilization chemotherapy with cyclophosphamide 4 g/m^2^ which was applied on two consecutive days (Table 2). During 2012, the intensity of the mobilization chemotherapy was reduced to cyclophosphamide 2 g/m^2^ with sufficient yield of the stem cell harvest. Three patients received plerixafor to increase the yield of harvested stem cells. CD34 columns were used in 76 grafts to concentrate hematopoietic stem cells and to remove potentially autoreactive lymphocytes from the graft.

Various protocols were used for conditioning chemotherapy according to the local standards (Table 3). The protocol of the ASTIS trial was considered as the standard protocol because the majority of transplant centers also participated in ASTIS [10].

**Table 3:** The protocols used for mobilization of hematopoietic stem cells, graft manipulation, and conditioning chemotherapy

Mobilization		Manipulation	Conditioning		
Cyc g/m^2^	Plerixafor	CD34 enrichment	Cyc or Mel	Thiotepa	TG or ATG
37 × 4 g/m^2^	-	76 × yes	60 × Cyc 200 mg/kg	-	39 × ATG 15–40 mg/kg
6 × 3 g/m^2^	1×	4 × no	10 × Cyc 100 mg/kg	10 × Thio 10 mg/kg	31 × TG 5–7.5 mg/kg
36 × 2 g/m^2^	2×		10 × Mel 200 mg/m^2^	-	10 × none
1 × 1 g/m^2^	-			-	

### SSc disease duration and age at HSCT

The age of the participants is usually restricted in clinical trials. We analyzed patients, who were younger than 50 years (mean 38.5 years, 76%) or older than 50 years (mean 57.3 years, 24%) when HSCT was performed (Table 4 part A). Older patients had a higher risk for ILD and PAH (33% versus 9%, *p*=0.025). The overall survival decreased from 95 to 83% when HSCT was performed before or after the age of 50 years (Table 4 part A). This suggested a higher mortality in patients with the diagnosis of SSc after age 50 years and rapidly progressive disease. Only 6 deaths (7.5%) were observed in this cohort of 80 patients with HSCT during the follow-up between mean 4.3 and 6.9 years (Table 4 part A).

**Table 4:** Patients in the HSCT group were predominantly treated before the age of 50 years (A). Patients with primary rapid progressive SSc were treated with HSCT within the first 3 years of the disease (B). Patients treated after 3 years had comparable survival rates after about 5 to 7 years of follow-up

**A**	**Age at HSCT<50y**	**Age at HSCT≥50y**		**B**	**Time Dg to HSCT <3y**	**Time Dg to HSCT ≥3y**	
n	57 (76%)	18 (24%)	p		43 (56%)	34 (44%)	p
female	32 (56%)	9 (50%)	0.787		18 (42%)	23 (68%)	0.035
male	25 (44%)	9 (50%)			24 (56%)	10 (29%)	
Age at Dg (mean ± SD)	33.9 ± 8.9	52.9 ± 5.7	<0.001		41.1 ± 11.6	35.0 ± 10.9	0.018
Age at HSCT	38.5 ± 8.2	57.3 ± 3.7	n.a.		43.2 ± 11.6	42.9 ± 10.2	0.949
Time Dg to HSCT	4.2 ± 4.8	3.8 ± 4.6	0.794		1.6 ± 0.8	7.4 ± 5.6	n.a.
diffuse cutaneous SSc	52 (91%)	14 (78%)	0.160		41 (95%)	27 (79%)	0.042
mRSS at HSCT	17.7 ± 11.7	19.4 ± 12.7	0.579		20.8 ± 11.5	14.5 ± 11.4	0.015
DLCO-SB at HSCT	56.0 ± 15.9	46.8 ± 18.4	0.261		52.3 ±17.0	55.6 ± 19.4	0.567
fVC at HSCT	73.8 ± 21.4	81.0 ± 18.4	0.261		76.1 ± 16.5	75.4 ± 24.8	1.000
ILD with PAH n(%)	5 / 56 (9%)	6 / 18 (33%)	0.024		7 / 42 (17%)	4 / 34 (12%)	0.328
Heart involvement	12 / 56 (21%)	7 / 18 (39%)	0.213		15 / 42 (36%)	4 / 34 (12%)	0.019
Time HSCT to last f-up	6.9 ± 4.8	4.3 ± 4.2	0.025		6.2 ± 4.7	7.0 ± 5.4	0.652
Alive at last f-up	54 / 57 (95%)	15 / 18 (83%)	0.145		38 / 43 (88%)	33 / 34 (97%)	0.220
Dead at last f-up	3 / 57 (5%)	3 / 18 (17%)			5 / 43 (12%)	1 / 34 (3%)	

Another restriction in clinical trials is the definition of early disease, which is usually defined as 3 years from the first non-Raynaud symptom to study baseline. In our HSCT cohort, 56% of patients were treated earlier than 3 years (mean 1.6 years) with HSCT compared to 44% of patients later than 3 years (mean 7.4 years, Table 4 part B). Since the age at HSCT was comparable in both groups (43.2 versus 42.9 years), this showed that a rapid progression of skin sclerosis usually occurs in older patients who are rapidly referred to an HSCT center. Five patients died in the HSCT group earlier than 3 years and 1 when HSCT was performed later than 3 years of SSc diagnosis. These findings confirm the strong selection of referral and HSCT centers for patients with multiple risk factors for rapidly progressive disease and considerable risk of TRM.

### Survival analyses

**Fig. 1:**
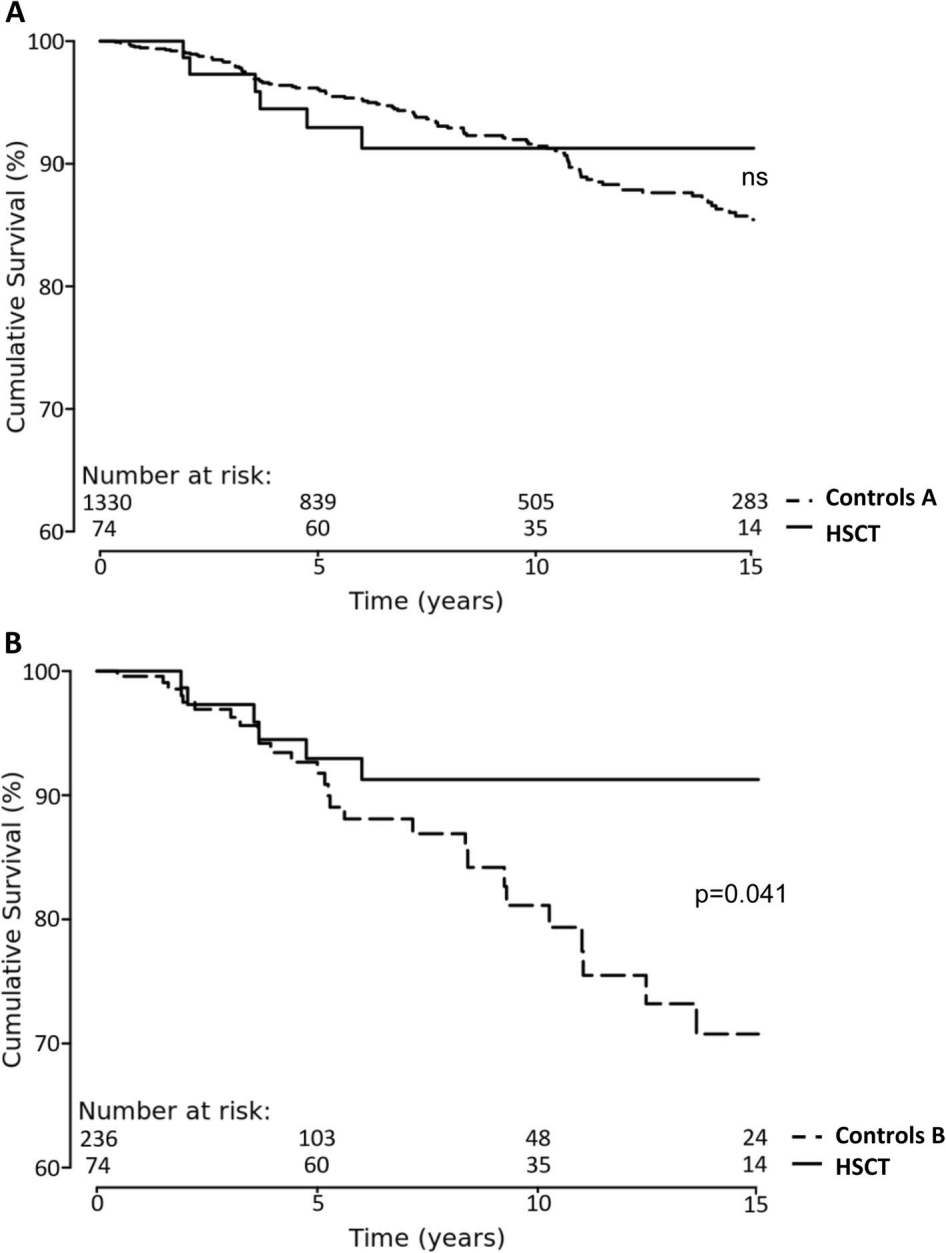
Overall survival of patients with HSCT compared to all dc-SSc patients in control group A (**A**) and compared to male dc-SSc patients positive for Scl70-antibodies (**B**). The observational period comprised 15 years of follow-up. Absolute numbers were indicated above the *x*-axis

The DNSS registry comprises long-term follow-up of SSc patients for up to 15 years (Fig. 1). A cumulative survival of 92% of HSCT patients was observed after 15 years of follow-up. Patients who did not receive HSCT seemed to live longer within the first 5 to 10 years after SSc diagnosis (Fig. 1A). The survival seemed to remain stable in HSCT and seemed to decline continuously in control group A and control group B during the 15 years of follow-up. However, the absolute numbers were getting small at the end of the long-term follow-up as indicated (Fig. 1). A more rapid decline of cumulative survival was observed in control group B (Fig. 1B). The 5-, 10-, and 15-year survival rates were 96%, 92%, and 86% in control group A (Fig. 1A) and 93%, 81%, and 71% in control group B which was significant after 15 years (*p*=0.041, Fig. 1B). The decline of the survival within the first 5 years of observation is associated with rapidly progressive SSc but not with HSCT or other treatments (Fig. 1B). The decline of patient numbers after 5-, 10-, and 15 years seemed to be only marginally attributed with a loss to follow-up. In the combined control group A and HSCT cohort, only 15 (3.0%), 25 (3.0%), and 41 (3.7%) patients were actually registered as lost to follow-up.

### Transplant-related and overall mortality

In the HSCT group, one of 80 patients died within 100 days after HSCT resulting in a TRM of 1.3% in this cohort. Additional 5 patients died later than 100 days post-HSCT due to progressive SSc. If we consider these patients as non-responders, we find a non-response rate of 6.3% after HSCT. This has to be compared with 125 patients (10.4%) who died in control group A and 31 patients (12.9%) who died in control group B.

### Skin sclerosis and pulmonary function after HSCT

Since the absolute numbers declined during follow-up, we focused on the first 5 years of follow-up in the DNSS registry. Because of an average delay of about 5 years between diagnosis and DNSS entry, the follow-up of years 0 to 5 in Table 3 refers to the years 5 to 10 after SSc diagnosis. We observed a significant decline of mRSS of 17.6 (± 11.5) to 11.0 (± 8.5) during the following 5 years. Patients in the high-risk control group B had a higher mRSS and lower DLCO-SB at baseline without a significant improvement during follow-up for 5 years (Table 5).

**Table 5:** The 5-year follow-up parameters for skin and lung involvement were indicated for HSCT and control groups A and B. The body mass index (BMI) was the lowest in the HSCT group and remained stable over the following 5 years

**Follow-up**	**Year 0**	**Year 1**	**Year 3**	**Year 5**
**all dc-SSc controls A (n=1513)**				
mRSS (mean ± SD)	15.6 ± 9.9	12.8 ± 8.8	12.1 ± 8.5	12.4 ± 9.0
N valid; p-value*	1242; reference	594; <0.001	361; <0.001	299; 0.191
DLCO SB (%) (mean ± SD)	63.2 ± 21.9	63.0 ± 21.8	61.2 ± 20.0	60.7 ± 20.3
N valid; p-value*	872; reference	455; 0.015	256; <0.001	216; <0.001
BMI (kg/m2) (mean ± SD)	24.3 ± 4.6	24.5 ± 4.8	24.2 ± 4.6	24.3 ± 4.8
N valid; p-value*	758; reference	402; 0.577	311; 0.971	285; 0.417
**male Scl70+ dc-SSc controls B (n=240)**				
mRSS (mean ± SD)	17.2 ± 9.7	14.2 ± 12.0	15.9 ± 9.0	15.5 ± 9.0
N valid; p-value*	218; reference	93; 0.050	45; 0.581	40; 0.957
DLCO SB (%) (mean ± SD)	59.1 ± 21.3	58.3 ± 23.7	56.7 ± 22.6	58.2 ± 25.3
N valid; p-value*	163; reference	78; 0.135	29; 0.192	35; 0.032
BMI (kg/m2) (mean ± SD)	25.0 ± 4.5	24.8 ± 4.1	25.0 ± 3.5	25.0 ± 4.1
N valid; p-value*	142; reference	68; 0.373	41; 0.948	38; 0.258
**HSCT SSc (n=79)**				
mRSS (mean ± SD)	17.6 ± 11.5	12.1 ± 8.6	13.6 ± 8.7	11.0 ± 8.5
N valid; p-value*	72; reference	57; <0.001	34; 0.006	33; 0.001
DLCO SB (%) (mean ± SD); p	54.6 ± 17.8	61.2 ± 21.0	55.6 ± 15.6	60.0 ± 18.5
N valid; p-value*	55; reference	47; 0.222	24; 0.980	20; 0.964
BMI (kg/m2) (mean ± SD); p	22.9 ± 4.1	23.0 ± 4.6	22.6 ± 3.9	22.8 ± 3.6
N valid; p-value*	56; reference	47; 0.844	33; 0.727	32; 0.175

If we consider a minimal detectable mRSS improvement of −2 points, we find at least minimal improvement of skin sclerosis in 48.0% of patients 1 year after HSCT. In control group B, we detected a minimal skin improvement in 28.4% of patients not earlier than 5 years after baseline (data not shown). These data suggest that 52.0% of patients do not show improvement of skin sclerosis or were transplanted for other reasons, e.g., heart or lung disease or an accumulation of several poor risk factors. If the response to HSCT was defined as a detectable improvement of skin sclerosis, and not only reduction of cumulative risk, we have to accept that about 30% of patients will not achieve an improvement of skin sclerosis (data not shown). Results from clinical trials estimated a minimally important difference in mRSS score in the range between 3.2 and 5.3 [18]. If we consider an mRSS response of −4 points as clinically relevant improvement, the mRSS response to HSCT would be lower than 40%.

## Discussion

In this retrospective registry analysis of more than 5000 patients with SSc, we demonstrated the efficacy of HSCT with regard to long-term overall survival in patients with severe SSc. The standard comparator group A was defined as all patients with dc-SSc. However, there were still significant differences in the distribution of risk factors for progressive SSc between the HSCT and group A. Male gender with diffuse cutaneous skin involvement and positivity for Scl70-antibodies are known to have a high risk of disease-related mortality [7]. Therefore, we defined an alternative comparator group B by adjusting for these risk factors. Despite our attempt to create appropriate control groups with a comparable distribution of risk factors, there were still significant differences between both control groups and the HSCT group, which is a limitation of this study. Analyses of matched pair controls are planned in the future in order to further homogenize the distribution of risk factors within the HSCT and control cohort. In this study, both control groups comprised a significant proportion of patients with lung and heart involvement, but DLCO was significantly better in group A with all dc-SSc patients but not in the Scl70+ male control group B, indicating that the Scl70+ male control group was a more appropriate comparator for the HSCT group. When we compared the HSCT group with the male Scl70+ group, the HSCT patients showed significantly better survival over time. This is in line with the results from three randomized controlled trials, which all showed a significant better overall and event-free survival in HSCT-treated patients when compared to a cyclophosphamide pulse therapy [9–11].

The benefit of this observational registry is its real-life character without the restrictions of inclusion or exclusion criteria. The long-term follow-up of our study and the large number of control patients are the major advantages of a longitudinal registry like DNSS compared to interventional trials.

The decline of patient numbers after 5, 10, and 15 years of follow-up is a limitation of this study. We cannot exclude the possibility that patients with a poor performance status were more likely to be lost to follow-up and the organ function of the remaining cohort seems to improve. However, this scenario seems to be unlikely because our results are consistent with the results from previously reported RCTs [9, 10]. As absolute patient numbers become very low over time, we focused on the 5-year follow-up and here we were able to observe a decline of survival within the first 5 years associated with rapidly progressive disease but not with HSCT or other treatment. Thus, HSCT was not a risk factor for early mortality but reduced the risk for mortality over the first 5 years. In addition, mRSS, as a marker for the skin involvement, significantly declined after HSCT. Another limitation of this study is the low number of documented fVC during the follow-up visits. However, the DLCO-SB suggests a stabilization of the lung function up to 5 years after HSCT.

With this knowledge and considering the rapid progress in the development of new therapies in SSc, every SSc patient should be referred to an experienced SSc center offering the opportunity to discuss all new therapeutic strategies including clinical trials and HSCT at an early stage of disease. There are several novel treatment options including antifibrotic and specific anti-inflammatory agents in development. However, up to now, these treatments cannot replace classic immunosuppressive therapy and HSCT in patients with rapidly progressive disease [9–11].

HSCT protocols have made progress over the years that reduced the risk of TRM and increased the acceptance of this treatment option. Still HSCT should only be performed at an experienced center. Although our patients have been transplanted between 1999 and 2021, we were unable to identify a difference between TRM in the early years compared with transplants in the recent years due to the small numbers of TRM.

Our observational study includes 80 transplanted patients. Therefore, it is one of the largest studies following HSCT treatment in patients with SSc [12]. We observed a very low HSCT-associated transplant-related mortality (TRM) of only 1.3% when compared with previous HSCT trials [9–11, 19, 20]. Nine patients received HSCT between 1997 and 2003, which was before the DNSS registry was initiated. Patients who received HSCT in the same period and died before 2003 would not be included in the DNSS registry, which could add as recruitment bias. Although we tried to select controls with a similar distribution of risk factor, we cannot exclude a potential selection bias in an open registry. Furthermore, some patients were lost to follow-up at some time. The lost to follow-up rate was indicated to be 3.0–3.7% after 5, 10, and 15 years. We cannot clearly differentiate between delayed follow-up reports and real lost to follow-up patients. Thus, we probably underestimate the real lost to follow-up rate to some extent. The delay of reporting deaths could have added to the low TRM in our cohort. Therefore, an HSCT TRM of 1.3% in our cohort was probably underreported. We can only speculate that the real HSCT TRM is likely rather between 1.3 and 5% when selected patients were treated in specialized HSCT centers.

Recently, Spierings et al. examined the correlation between HSCT RCT inclusion and exclusion parameters with survival as an outcome in an observational SSc cohort not treated by HSCT, but standard immunosuppression [21]. They showed that patients who meet the RCT inclusion criteria had a particularly poor outcome. Those patients who also meet HSCT exclusion criteria like age, pulmonary hypertension, poor kidney function, or DLCO <40% had an even worse survival [21]. This defines a group of patients with poor prognosis who might have benefitted from HSCT treatment, but also a group with even worse prognosis in which treatment options are very limited and for which there is a high unmet need. Further studies of therapeutic options for these high-risk patients are warranted.

Another issue is the definition of early progressive SSc, which should be considered for HSCT. Between the late 1990 years and 2012, TRM rates between 10 and 17% were reported [10, 19]. After 2012 and probably due to standardized selection of SSc patients, the TRM rates were around 7% [10, 19]. Selection to early SSc means an SSc duration of less than 2 years and clinically progressive skin sclerosis and lung fibrosis before HSCT. Patients with longer SSc duration were considered to accumulate advanced lung fibrosis, heart involvement, and pulmonary arterial hypertension that would contribute significantly to a higher TRM after 4 years of SSc duration. Our data show that HSCT is feasible up to 7 years after the onset of SSc and TRM is not increased in these patients. Our data also show that selection of SSc patients in HSCT referral centers reduced toxicities of mobilization and conditioning regimens and advances in the supportive care probably contribute to lower TRM rates below 7%.

In summary, our results on long-term survival after HSCT from our large DNSS SSc registry demonstrate a benefit of HSCT in a selected patient cohort with a high risk for rapidly progressive disease. Here, Scl70 positivity, dc-SSc, and male gender were the evaluated risk factors but there are even more risk factors that we know from other studies [7, 20, 22, 23]. Further analyses of risk factors in early SSc disease should define those patients who probably benefit the most from an early intensive intervention like HSCT. Further analyses should also compare the risk of HSCT TRM with the risk of patients with early progressive SSc who meet exclusion criteria for HSCT treatment.

## Data Availability

Not applicable.
